# Methionine and Glycine Stabilize Mitochondrial Activity in Sake Yeast During Ethanol Fermentation

**DOI:** 10.17113/ftb.57.04.19.5665

**Published:** 2019-12

**Authors:** Jannatul Ferdouse, Yuki Kusaba, Yuki Fujimaru, Yuki Yamamoto, Hiroshi Kitagaki

**Affiliations:** 1 Department of Environmental Science, Faculty of Agriculture, Saga University, Saga City, Saga 840-8502, Japan; 2 Department of Biochemistry and Applied Biosciences, United Graduate School of Agricultural Sciences, Kagoshima University, 1-21-24, Korimoto, Kagoshima 890-8580, Japan; 3 Department of Microbiology, Faculty of Biological Sciences, University of Chittagong, Chittagong 4331, Bangladesh

**Keywords:** sake yeast, ethanol, fermentation, amino acids, mitochondria

## Abstract

Addition of amino acids to fermentation media affects the growth and brewing profiles of yeast. In addition, retaining mitochondrial activity during fermentation is critical for the fermentation profiles of brewer’s yeasts. However, a concrete mechanism linking amino acids in fermentation media with mitochondrial activity during fermentation of brewer’s yeasts is yet unknown. Here, we report that amino acids in fermentation media, especially methionine (Met) and glycine (Gly), stabilize mitochondrial activity during fermentation of sake yeast. By utilizing *atg32△* mutant sake yeast, which shows deteriorated mitochondrial activity, we screened candidate amino acids that strengthened the mitochondrial activity of sake yeast during fermentation. We identified Met and Gly as candidate amino acids that fortify mitochondrial activity in sake yeast during fermentation. To confirm this biochemically, we measured reactive oxygen species (ROS) levels in sake yeast fermented with Met and Gly. Yeast cells supplemented with Met and Gly retained high ROS levels relative to the non-supplemented sake yeast. Moreover, Met-supplemented cells showed a metabolome distinct from that of non-supplemented cells. These results indicate that specific amino acids such as Met and Gly stabilize the mitochondrial activity of sake yeast during fermentation and thus manipulate brewing profiles of yeast.

## INTRODUCTION

The nutrients in fermentation media affect the characteristics of brewer’s yeasts. For example, assimilable nitrogen increases the growth rate of yeast (*1*). Furthermore, the response of yeast cells to various amino acids differs (*2*-*6*). Specifically, yeasts supplemented with methionine (*7*, *8*), biotin, pantothenic acid and vitamin B6 (*9*, *10*) show decreased production of hydrogen sulfide, which produces an off-flavour (*11*, *12*). However, a concrete mechanism underlying the effects of exposure to these amino acids remains to be elucidated.

The mitochondrion is an organelle essential for oxidative respiration. Molecular oxygen breaks down rapidly upon fermentation, leading to rapid loss of mitochondrial activity. However, we and other groups have reported that residual mitochondrial activity plays significant roles in the fermentation characteristics of brewer’s yeasts, such as hydrogen sulfide formation (*13*), diacetyl formation (*14*), fermentation ability (*15*), volatile ester formation (*16*), fatty acid desaturation (*17*), and malate and succinate production (*18*). Furthermore, we have previously shown that mitophagy, the degradation of mitochondria by autophagy (*19*-*22*), plays a significant role in the progression of alcoholic fermentation (*15*), as observed by the high fermentation ability of *atg32Δ* sake yeast. Therefore, the mitochondrial activity of brewer’s yeasts plays critical roles in their fermentation profiles.

Until now, oxygenation has been the only approach to manipulate the mitochondrial activity of brewer’s yeasts during fermentation. In this study, we attempted to develop a method of manipulating the mitochondrial activity of brewer’s yeasts during fermentation by exploring the mechanism underlying the effect of amino acids on mitochondrion-related phenomena during fermentation. By adopting a system to detect mitochondrion-activating substances, we screened for amino acids that could modify mitochondrial activity during fermentation. As a result, we identified methionine (Met) and glycine (Gly) as candidates to activate mitochondrial activity. Consistent with the hypothesis, these amino acids strengthened the mitochondrial activity, as detected by reactive dye 2,7-dichlorodihydrofluorescein diacetate (DCFH-DA), which binds to the reactive oxygen species. Reactive oxygen species (ROS) are byproducts of the mitochondrial respiratory chain in aerobically growing cells (*23*) that may contribute to intracellular oxidative stress. However, previous studies indicated that ROS production also occurs during fermentation (*24*). Under anaerobic conditions, yeast activates cytochrome P450 systems which produce significant levels of ROS products such as H_2_O_2_ (*25*). Since the increase of ROS formation causes the increase of mitochondrial respiration and its energy coupling (*26*), we analyzed the mitochondrial activity by measuring intracellular ROS production. These results indicate that specific amino acids stabilize mitochondrial activity during fermentation, indicating a new mechanism linking amino acid supplementation and the fermentation profiles of brewer’s yeast.

## MATERIALS AND METHODS

### Yeast strains and media

Sake yeast RAK1536 *MAT***a/α** *his3/his3* (*27*) and its mutant RAK1536 *atg32△::kanMX/atg32△::NAT1* (*15*) were used in this study. The yeast cells were propagated in yeast extract-peptone-dextrose (YPD) medium containing 2% (*m*/*V*) Bacto peptone, 1% (*m*/*V*) Bacto yeast extract (Becton Dickinson, Franklin Lakes, NJ, USA), and 2% (*m*/*V*) glucose. For the fermentation tests, a minimal synthetic medium containing 0.67% (*m*/*V*) yeast nitrogen base without amino acids (Becton Dickinson), 790 mg/L complete supplement mixture (CSM) Dropout: Complete (Formedium, Hunstanton, UK), and 15% (*m*/*V*) glucose with amino acids was used.

### Fermentation test

Yeast cells were inoculated in 60 mL of minimal synthetic medium containing 2% (*m*/*V*) glucose in a 100-mL flask. The flask was incubated at 30 °C overnight under aerobic and shaking conditions at 200 rpm for pre-enrichment culture. Then, yeast cells (10^6^ cells/mL) were inoculated in 100 mL minimal synthetic medium containing 15% (*m*/*V*) glucose with or without amino acids in a 300-mL Erlenmeyer flask equipped with an air lock at the top, and cultured statically at 30 °C for 9 to 11 days. The culture mass was determined daily.

### Measurement of ethanol volume fraction

After fermentation, 1-mL sample was collected from fermentation media and centrifuged (model 5200; KUBOTA Co. Ltd, Osaka, Japan) at 7000×*g* for 1 min. After centrifugation, the supernatant was collected and kept on ice until ethanol measurement. The ethanol volume fraction of fermented media was analyzed using a contact combustion system with an alcohol densitometer (alcohol checker YSA-200; Yazaki Meter Co. Ltd., Tokyo, Japan) according to the manufacturer’s instructions, as described previously (*28*). Standard ethanol volume fraction was maintained with 15% ethanol.

### Measurement of glucose mass fraction

Glucose mass fraction was measured with a Glucose CII-kit (Wako Pure Chemical Industries Ltd., Osaka, Japan) at 505 nm using a UV spectrophotometer (UV-1800; Shimadzu Scientific Instruments, Kyoto, Japan) as described previously (*29*). Briefly, after the fermentation, the supernatant was collected by centrifugation (model 5200; KUBOTA Co. Ltd) at 7000×*g* for 1 min. Then 20-µL sample was mixed with 3 mL of colour reagent and incubated for 5 min at 37 °C. The absorbance was then measured at 505 nm. Standard solution was also prepared in the same way, the absorbance was measured and glucose mass fraction was calculated from the calibration curve.

### Measurement of dry cell mass

After fermentation, cells were collected by centrifugation (model B51057; KUBOTA Co. Ltd) at 3200×*g* for 10 min and washed twice with sterile water, suspended in 1 mL sterile water, and added to 250-mL aluminium bottles that were weighed before adding the cells. The bottles with cells were then heated overnight at 100 °C and weighed again. The differences between the mass of the bottles before and after the addition of cells were presented as the dry cell mass, which was expressed as biomass.

### Analysis of intracellular ROS content

2,7-Dichlorodihydrofluorescein diacetate (DCFH-DA; Funakoshi, Tokyo, Japan) stock solution of 1 mg was prepared in 100 µL ethanol and stored at -20 °C. Yeast cells (10^6^ cells/mL) were inoculated in 10 mL minimal synthetic medium containing 15% (*m*/*V*) glucose with or without amino acids and incubated for 2–3 days at 30 °C with liquid paraffin overlaid on the culture. After fermentation, 10^7^ cells/mL yeast cells were recovered and incubated with 13 µL DCFH-DA, 100 µL phosphate-buffered saline (PBS), pH=7.4, and 887 µL sterile water at 30 °C for 20 min in a shaker (H500-H; Benchmark Scientific Inc., Sayreville, NJ, USA) at 200 rpm. After incubation, the cells were washed three times with sterile water, centrifuged (KUBOTA 5200; Kubota, Co. Ltd.) at 13 000×*g* for 1 min and 100 cells were sampled under fermentation stress, treated with DCFH-DA and the ROS content was analyzed by fluorescence microscopy (Olympus BX53; Olympus, Tokyo, Japan).

### Metabolite analysis by GC/FID method

After fermentation, the sample pellet was collected by centrifugation at 3200×*g* and at -9 °C for 3 min, then resuspended in 3 mL MilliQ water (Millipore Inc., Darmstadt, Germany) and centrifuged again under the same conditions. The pellet was then soaked in liquid nitrogen for 3.5 min, freeze-dried for 12 h, and stored at -80 °C. Next, 10 mg of the dried pellet were mixed with a mixture of chloroform, methanol (both Kanto Chemical Co., Tokyo, Japan) and water at ratio 2:5:2 for extraction, followed by the addition of 60 µL ribitol (0.2 mg/mL, Wako Pure Chemical Industries Ltd.) to the solvent, and incubation for 3 min at 30 °C with shaking at 1500 rpm. Then, 800 µL supernatant were collected after centrifugation (4 °C and 16 000×*g* for 3 min) and 400 µL MilliQ water (Millipore Inc) were added, mixed and centrifuged again under the same conditions. We then evaporated 800 µL of the supernatant for 3 h and freeze-dried it for 12 h. Next, 100 µL methoxyamine (20 mg/mL dissolved in pyridine, Nacalai Tesque, Kyoto, Japan) were mixed with the freeze-dried extract and incubated at 30 °C for 90 min with shaking at 1500 rpm, followed by the addition of 50 µL *N*-methyl-*N*-(trimethylsilyl) trifluoroacetamide (MSTFA) and incubation at 37 °C and 1500 rpm for 30 min with shaking. Then, 70 µL of the solvent were transferred to a vial and the metabolites were analyzed using a gas chromatograph with flame ionization detector (GC-2014; Shimadzu) with a CP Sil8CB column (30 m×0.25 mm×0.25 µm; Agilent Technologies, Palo Alto, CA, USA). The data were analysed with Lab Solutions v. 5.71 SP1 (*30*) and SIMCA v. 13.0 (*31*). Nitrogen was used as the carrier gas, with a column headspace pressure of 73.9 kPa and a flow rate of 0.97 mL/min. The gas chromatography temperature program was as follows: 60 °C for 2 min, raised to 320 °C at 13 °C/min and held for 17 min. The split ratio for intracellular metabolites was 2. The metabolites were analyzed using GC/FID (Lab Solutions software) and their peak area was divided by the total peak area, and standardized by auto-scaling. Experiments were performed in triplicate with independent cultures.

### Statistical analysis

The statistical significance of the differences between average values of two or more data groups was determined using Dunnett’s test without known deviations. The experimental results were expressed as mean value ± standard error of the mean. The results were considered significant when p<0.05. Statistical significance of the interaction was also determined using 2-way ANOVA.

## RESULTS AND DISCUSSION

### ROS analysis of defective mitochondria of the atg32△ sake mutant yeast

We first hypothesized that *atg32△* sake mutant yeast showed high fermentation ability (*15*) because of defective mitochondria. To prove this hypothesis, the levels of reactive oxygen species (ROS) in the *atg32△* sake mutant yeast were analyzed using DCFH-DA. The *atg32△* sake mutant yeast exhibited significantly (p*<*0.05, Dunnett’s test) weak reactive oxygen species activity compared to the parental strain. The fluorescence intensity of sake mutant *atg32△* was 30.2±0.4 and of its parental strain was 34.7±0.7, indicating that the *atg32△* sake mutant yeast has defective mitochondrial activity. It was thus considered that *atg32△* sake mutant yeast shows high ethanol fermentation ability (*15*) because *atg32△* has an elevated cytosolic carbon flux due to its deteriorated mitochondrial function caused by disrupting mitophagy. This led us to conclude that loss of mitochondrial activity during ethanol fermentation increases fermentation ability, *i.e.* augmentation of mitochondrial activity decreases fermentation ability.

### Identification of methionine and glycine as mitochondrion-activating amino acids

To identify the amino acid that activates mitochondrial activity during fermentation, we invented a system using *atg32△* sake mutant yeast. We hypothesized that augmentation of mitochondrial activity could be detected by comparing the ratio of increasing fermentation ability of wild-type and mutant *atg32△* sake yeasts. In wild-type yeast, the addition of amino acids increased the fermentation ability as a consequence of the increase of the protein content in the cytosol, leading to the acceleration of glycolysis. This should be the same in *atg32△* sake mutant yeast, but its fermentation ability was counteracted by the increase of mitochondrial activity. Based on this scheme, we examined the effect of amino acids on the fermentation abilities of sake yeast (Table 1 and Table 2). As a result, amino acids such as methionine (Met), cysteine (Cys), glycine (Gly), proline (Pro), phenylalanine (Phe), leucine (Leu), isoleucine (Ile), tyrosine (Tyr), tryptophan (Trp), serine (Ser), glutamine (Gln), histidine (His) and aspartic acid (Asp) showed the above characteristics in CO_2_ evolution (g/L). In terms of maximum fermentation rate, amino acids such as Met, Cys, Gly, Ala, Val, Pro, Ile, Tyr, Ser and Lys were effective (g/(L·day)). Moreover, in terms of final ethanol volume fraction, amino acids such as Met, Cys, Gly, Pro, Leu, Tyr, Trp, Ser, Gln, Lys, Arg, His and glutamic acid (Glu) showed similar characteristics. To examine this, we analyzed the growth profile (*A*_600 nm_·10^7^ cells/mL) of wild-type and sake mutant *atg32△* yeasts with or without the addition of amino acids. As for growth profile, Met, Cys, Gly, Val, Pro, Tyr, Ser, Gln, Arg and Asp showed similar effects in this study. The above results suggested that Met, Cys, Gly, Pro, Tyr and Ser showed common significant interaction effects for main fermentation characteristics among all amino acids (Table 3). In Table 3, we showed the interaction effects of wild type (WT) and *atg32△* with or without amino acids by calculating slope values from Table 1 and Table 2 data. If the slope ratio between WT and *atg32△* with or without amino acids is high or negative (not parallel), they have significant interaction (p*<*0.05, two-way ANOVA). On the other hand, if the slope ratio is very low and positive (parallel), they do not have significant interaction.

**Table 1:** Fermentation characteristics of wild-type (WT) sake yeast cells cultured in minimal synthetic medium containing 15% glucose with or without amino acids

Sample no.	*γ*(CO_2_)/(g/L)		Max. fermentation rate /(g/(L·day))		*φ*(ethanol)/%	
Average	Fold change	Average	Fold change	Average	Fold change
WT	63.9±0.3	1.00	14.5±0.1	1.00	8.58±0.02	1.00
WT+Met	64.3±0.2	1.01	(15.6±0.1)*	1.07	8.65±0.02	1.01
WT+Cys	(68.3±0.2)*	1.07	(15.7±0.1)*	1.08	(8.75±0.02)*	1.02
WT+Gly	64.7±0.1	1.01	(15.5±0.2)*	1.07	(8.75±0.00)*	1.02
WT+Ala	(66.9±0.3)*	1.05	(15.5±0.1)*	1.06	8.72±0.03	1.02
WT+Val	(67.9±0.4)*	1.06	(15.7±0.1)*	1.08	(8.77±0.03)*	1.02
WT+Pro	(66.8±0.2)*	1.04	(15.7±0.2)*	1.08	(8.73±0.01)*	1.02
WT+Phe	(65.5±0.4)*	1.02	15.1±0.2	1.04	8.57±0.03	1.00
WT+Leu	(67.0±0.1)*	1.05	(15.5±0.1)*	1.06	(8.73±0.03)*	1.02
WT+Ile	(65.5±0.2)*	1.02	15.0±0.2	1.03	8.55±0.05	1.00
WT+Tyr	(66.7±0.2)*	1.04	(15.5±0.1)*	1.07	(8.75±0.02)*	1.02
WT+Trp	(66.3±0.2)*	1.03	(15.3±0.2)*	1.05	8.68±0.01	1.01
WT+Ser	(67.0±0.2)*	1.05	(15.6±0.1)*	1.07	(8.73±0.03)*	1.02
WT+Thr	(65.9±0.2)*	1.03	(15.7±0.1)*	1.08	(8.75±0.02)*	1.02
WT+Gln	(65.9±0.2)*	1.03	(15.5±0.1)*	1.07	(8.73±0.01)*	1.02
WT+lys	63.0±0.2	0.99	15.0±0.1	1.03	8.63±0.01	1.01
WT+Arg	(67.1±0.3)*	1.05	(15.5±0.1)*	1.06	(8.78±0.03)*	1.02
WT+His	(67.1±0.2)*	1.05	(15.4±0.1)*	1.06	8.70±0.02	1.01
WT+Asp	(67.2±0.2)*	1.05	(15.7±0.1)*	1.08	(8.75±0.02)*	1.02
WT+Glu	(65.9±0.3)*	1.03	(15.5±0.1)*	1.07	(8.73±0.03)*	1.02
WT+Asn	(65.7±0.2)*	1.03	(15.4±0.1)*	1.06	8.71±0.01	1.01

Values are mean±SEM of three independent experiments with respective starter cultures. *p<0.05 as determined by Dunnett’s test. Met=methionine, Cys=cysteine, Gly=glycine, Ala=alanine, Val=valine, Pro=proline, Phe=phenylalanine, Leu=leucine, Ile=isoleucine, Tyr=tyrosine, Trp=tryptophan, Ser=serine, Thr=threonine, Gln=glutamine, Lys=lysine, Arg=arginine, His=histidine, Asp=aspartic acid, Glu=glutamic acid and Asn=asparagine

**Table 2:** Fermentation characteristics of the mutant *atg32△* sake yeast cells cultured in minimal synthetic medium containing 15% glucose with or without amino acids

Sample no.	*γ*(CO_2_)/(g/L)		Max. fermentation rate/ (g/(L·day))		*φ*(ethanol)/%	
Average	Fold change	Average	Fold change	Average	Fold change
*atg32△*	69.3±0.2	1.00	15.57±0.07	1.00	8.96±0.02	1.00
*atg32△*+Met	68.2±0.2	0.98	15.9±0.1	1.02	8.90±0.03	0.99
*atg32△*+Cys	(71.2±0.3)*	1.03	16.1±0.1	1.03	9.03±0.02	1.01
*atg32△*+Gly	69.20±0.06	1.00	16.0±0.1	1.02	9.03±0.02	1.01
*atg32△*+Ala	(71.0±0.3)*	1.02	15.8±0.1	1.01	9.02±0.02	1.00
*atg32△*+Val	(71.7±0.5)*	1.03	16.0±0.2	1.03	9.05±0.03	1.01
*atg32△*+Pro	68.8±0.1	0.99	15.9±0.1	1.02	8.98±0.02	1.00
*atg32△*+Phe	66.7±0.1	0.96	15.8±0.1	1.01	8.83±0.03	0.98
*atg32△*+Leu	69.2±0.3	1.00	(16.2±0.2)*	1.04	8.97±0.03	1.00
*atg32△*+Ile	66.6±0.2	0.96	15.4±0.2	0.99	8.80±0.06	0.98
*atg32△*+Tyr	69.0±0.2	0.99	15.9±0.1	1.02	8.98±0.02	1.00
*atg32△*+Trp	67.8±0.2	0.98	15.9±0.1	1.02	8.95±0.03	1.00
*atg32△*+Ser	69.3±0.1	1.00	15.9±0.2	1.02	9.00±0.03	1.00
*atg32△*+Thr	(71.2±0.1)*	1.03	(16.2±0.2)*	1.04	(9.10±0.03)*	1.01
*atg32△*+Gln	68.83±0.09	0.99	16.0±0.1	1.03	8.98±0.02	1.00
*atg32△*+lys	67.8±0.3	0.98	15.53±0.09	0.99	8.90±0.03	0.99
*atg32△*+Arg	(70.8±0.2)*	1.02	16.0±0.2	1.03	9.02±0.02	1.00
*atg32△*+His	69.8±0.1	1.01	16.0±0.1	1.03	8.98±0.02	1.00
*atg32△*+Asp	70.1±0.2	1.01	(16.3±0.2)*	1.05	(9.08±0.02)*	1.01
*atg32△*+Glu	(70.8±0.2)*	1.02	16.0±0.1	1.03	9.02±0.02	1.00
*atg32△*+Asn	(71.0±0.2)*	1.02	(16.2±0.2)*	1.04	9.03±0.02	1.01

Values are mean±SEM of three independent experiments with respective starter cultures. *p<0.05 as determined by Dunnett’s test. For amino acid abbreviations see Table 1

**Table 3:** The fold changes of the fermentation parameters of wild-type (WT) *vs* its mutant *atg32△* sake yeast cells cultured in minimal synthetic medium containing 15% glucose after the addition of amino acids (AA)

Sample no.	*γ*(CO_2_)/(g/L)		Max. fermentation rate/ (g/(L·day))		*φ*(ethanol)/%		Growth profile (*A*_600 nm_. 10^7^ cells/mL)	
Fold change (WT/*atg32Δ*)	p*-*value	Fold change (WT/*atg32Δ*)	p*-*value	Fold change (WT/*atg32Δ*)	p*-*value	Fold change (WT/*atg32Δ*)	p-value
WT/*atg32△*(Met)	0.43/-1.04	0.011	1.04/0.36	0.021	0.07/-0.06	0.022	-0.12/0.06	0.006
WT/*atg32△*(Cys)	4.43/1.90	0.001	1.20/0.53	0.029	0.17/0.07	0.039	-0.12/0.08	0.004
WT/*atg32△*(Gly)	0.77/-0.07	0.055	0.97/0.40	0.045	0.17/0.07	0.008	-0.08/0.09	0.022
WT/*atg32△*(Ala)	3.00/1.76	0.065	0.94/0.20	0.010	0.14/0.06	0.095	0.03/0.13	0.175
WT/*atg32△*(Val)	4.00/2.40	0.062	1.20/0.43	0.024	0.19/0.09	0.080	-0.06/0.07	0.049
WT/*atg32△*(Pro)	2.90/-0.50	0.001	1.20/0.36	0.007	0.15/0.02	0.009	-0.12/0.08	0.005
WT/*atg32△*(Phe)	1.60/-2.57	0.001	0.60/0.20	0.153	0.02/0.13	0.067	0.10/0.15	0.521
WT/*atg32△*(Leu)	3.10/-0.07	0.001	0.94/0.59	0.256	0.15/0.01	0.021	0.01/0.10	0.132
WT/*atg32△*(Ile)	1.56/-2.64	0.001	0.47/-0.14	0.051	0.03/0.16	0.133	0.05/0.15	0.131
WT/*atg32△*(Tyr)	2.83/-0.27	0.001	0.97/0.33	0.027	0.17/0.02	0.006	-0.13/0.08	0.003
WT/*atg32△*(Trp)	2.13/-1.50	0.001	0.74/0.36	0.196	0.10/-0.01	0.021	0.06/0.13	0.319
WT/*atg32△*(Ser)	3.07/-0.03	0.001	1.07/0.36	0.007	0.15/0.04	0.037	-0.09/0.08	0.016
WT/*atg32△*(Thr)	1.97/1.90	0.886	1.12/0.66	0.118	0.17/0.14	0.499	0.02/0.06	0.582
WT/*atg32△*(Gln)	1.93/-0.44	0.001	0.97/0.46	0.062	0.15/0.02	0.035	-0.01/0.10	0.052
WT/*atg32△*(Lys)	0.87/1.44	0.295	0.50/-0.04	0.040	0.05/-0.06	0.028	0.03/0.12	0.210
WT*/atg32△*(Arg)	3.20/1.53	0.061	0.94/0.43	0.095	0.20/0.06	0.011	-0.05/0.08	0.055
WT/*atg32△*(His)	3.17/0.56	0.001	0.90/0.43	0.062	0.12/0.02	0.047	0.02/0.09	0.269
WT/*atg32△*(Asp)	3.30/0.86	0.001	1.17/0.73	0.176	0.17/0.12	0.255	-0.04/0.07	0.052
WT/*atg32△*(Glu)	2.03/1.53	0.345	0.97/0.46	0.062	0.15/0.06	0.053	0.05/0.12	0.201
WT/*atg32△*(Asn)	1.80/1.73	0.892	0.90/0.60	0.282	0.13/0.07	0.080	0.05/0.08	0.736

The statistical significance of interaction was analyzed using two-way factorial ANOVA (p<0.05). For amino acid abbreviations see Table 1

### A*ddition of Met and Gly fortifies mitochondrial activity of sake yeast during fermentation*

**Fig 1:**
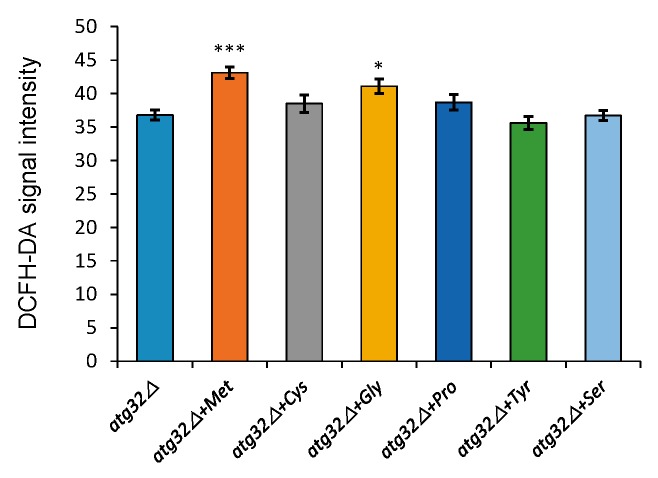
Evaluation of intracellular reactive oxygen species (ROS) content in the mutant *atg32△* sake yeast cells incubated in minimal synthetic medium containing 15% glucose with or without amino acids: methionine (Met), cysteine (Cys), glycine (Gly), proline (Pro), tyrosine (Tyr) and serine (Ser). The statistical significance of the difference is indicated by Dunnett’s test (***p<0.001, *p<0.05)

We hypothesized that amino acids Met, Cys, Gly, Pro, Tyr and Ser might fortify mitochondrial activity of mutant *atg32△* sake yeast during fermentation. To test this hypothesis, we measured the mitochondrial activities of *atg32△* sake mutant yeast supplemented with these amino acids by measuring the levels of reactive oxygen species (ROS) using DCFH-DA. Among these amino acids, only the addition of Met and Gly significantly (p*<*0.05) increased the fluorescence intensity of *atg32△* sake mutant yeast (Fig. 1) compared to that of sake yeast. Together with the above-mentioned results, these findings clearly indicate that Met and Gly function to stabilize the mitochondrial activity in *atg32△* sake mutant yeast.

### Metabolome analysis of sake yeast supplemented with Met

**Fig 2:**
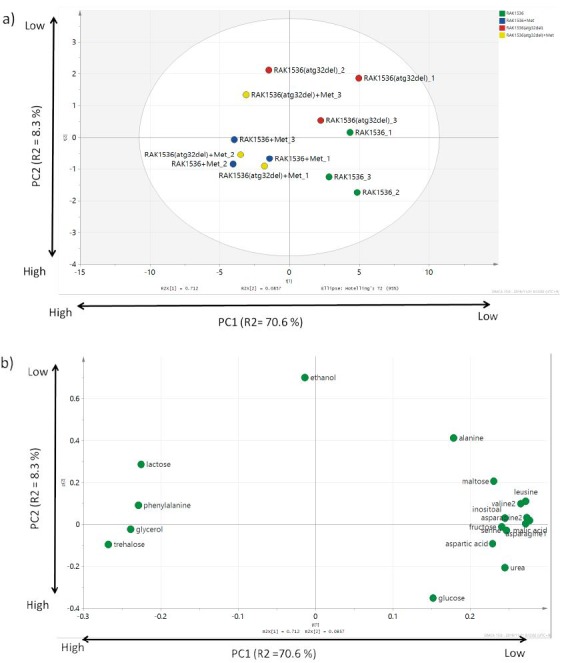
The intracellular metabolites of wild-type (RAK1536) and mutant *atg32△* sake yeast cells incubated in minimal synthetic medium containing 15% glucose with or without methionine (Met). Normalized values of measured peak areas were used as independent variable in PCA (p<0.05): a) score plot of PCA, and b) loading plot of PCA

To obtain information on the physiology of *atg32Δ* yeast cells supplemented with Met, we analyzed yeast metabolome. Yeast cells supplemented with Met were clearly separated from non-supplemented cells (Fig. 2a). Ethanol contributed to the separation of *atg32Δ* and glucose contributed to the separation of WT (Fig. 2b), consistent with the high fermentation ability of *atg32Δ*. Met-supplemented cells were clustered together, consistent with the similar fermentation ability of WT and *atg32Δ* (Table 3). Most of amino acids contributed negatively to the separation of Met-supplemented cells, suggesting that the glycolysis pathway, which generates simple substances, was promoted in Met-supplemented cells. Metabolome analysis revealed the high fermentation ability of WT and *atg32Δ* cells supplemented with Met as relative to non-supplemented cells in this study. However, the metabolome did not provide concrete information on the mitochondrial metabolite. This was because the metabolite content within mitochondria during fermentation was too low to detect. This hypothesis needs to be proven in further experiments.

Mitochondria maintain their transmembrane electron potential through the electron transport chain (*32*, *33*). Since the function of the electron transport chain is based on the function of iron, formation of iron-sulfur (Fe-S) proteins might be facilitated by specific amino acids (*34*, *35*). Alternatively, the amino acids used in this research, or organic acids arising from the supplemented amino acids (*36*), might have entered the mitochondria and stimulated the mitochondrial metabolism. The mechanism underlying mitochondrial activity upregulation requires further research. After Met is incorporated into yeast cells through Mup1 (*37*), it is used as a component of proteins and is also transaminated with α-ketoglutarate to α-keto-γ-methylthiobutyrate. It is also converted to methanethiol, methionol, and α-ketobutyrate in a reductive environment (*38*). These substances are suggested to stimulate the mitochondria. Consistent with this hypothesis, genes encoding mitochondrion-localized proteins were highly expressed in yeast cells supplemented with Met (*4*). It is also suggested that one electron pair from the sulfur atom of Met or its reductive form methionol might scavenge the ROS during anaerobiosis (*39*) and protect iron-sulfur clusters, thus stabilizing the mitochondria. The response of yeast cells supplemented with Met is similar to those supplemented with Leu, Ile, Thr, Trp and Tyr, which confer slow yeast growth (*4*). Together with the data obtained in our study, it is suggested that unfavourable amino acids like Met stimulate mitochondrial activity. However, the mechanism of mitochondrial stimulation with Met requires further investigation. Addition of Met leads to decreased production of hydrogen sulfide, which leads to an undesirable flavour (*8*). It can be postulated that iron-sulfur cluster formation in mitochondria (*34*) is enhanced and affects hydrogen sulfite production from sulfate, as reported previously (*13*). Therefore, mitochondrial stabilization by Met as observed in this study might provide a link between Met, mitochondria, and hydrogen sulfide production.

After Gly incorporation into yeast cells by the general amino acid permease Gap1 (*40*), it is used as a component of proteins and is also cleaved to amino or carboxyl moieties through the glycine cleavage system (*41*). Gly increased the mitochondrial activity of *atg32△* sake yeast in this study. The degradation products like methylene-THF (tetrahydrofolic acid) generated by the glycine cleavage system might have entered the mitochondria and activated their metabolism. The mechanism of the effect of Gly on the mitochondria of sake yeast needs further research.

The most abundant amino acids in sake are Asp, Arg, Pro, Ala, Gly, Glu and Leu (*42*). It has been reported that pantothenic acid decreases hydrogen sulfide in sake yeast (*43*). Increase in Pro confers ethanol resistance in sake yeast (*44*). The results obtained in this study suggest that the specific amino acids generated during sake brewing affect the mitochondrial activity of sake yeast and thus its brewing profiles. Since the regulation of mitochondria in brewer’s yeasts affects hydrogen sulfide formation (*13*), diacetyl formation (*14*), fermentation ability (*15*), volatile ester formation (*16*), fatty acid desaturation (*17*), and malate and succinate production (*18*), many brewers attempt to manipulate the mitochondrial activity of brewer’s yeasts by oxygenation. The results obtained in this study suggest that amino acid supplementation might act as a new approach to manipulate mitochondrial activity in brewer’s yeasts. Further pilot-level studies are needed to verify this technique.

## CONCLUSION

It has been accepted that mitochondria of brewer’s yeasts lose their activity as fermentation proceeds. By adopting the *atg32Δ* system and analyzing the fermentation profiles, we have revealed that methionine and glycine stabilize the mitochondrial activity of sake yeast. This new knowledge will be a new approach to stabilize mitochondria during fermentation.
